# Online supervised attention-based recurrent depth estimation from monocular video

**DOI:** 10.7717/peerj-cs.317

**Published:** 2020-11-23

**Authors:** Dmitrii Maslov, Ilya Makarov

**Affiliations:** 1 School of Data Analysis and Artificial Intelligence, HSE University, Moscow, Russia; 2 Samsung-PDMI Joint AI Center, St. Petersburg Department of Steklov Institute of Mathematics, St. Petersburg, Russia

**Keywords:** Computer Vision, Depth Reconstruction, Autonomous Vehicles, Augmented Reality, Deep Convolutional Neural Networks, Recurrent Neural Networks, Computer Science Methods

## Abstract

Autonomous driving highly depends on depth information for safe driving. Recently, major improvements have been taken towards improving both supervised and self-supervised methods for depth reconstruction. However, most of the current approaches focus on single frame depth estimation, where quality limit is hard to beat due to limitations of supervised learning of deep neural networks in general. One of the way to improve quality of existing methods is to utilize temporal information from frame sequences. In this paper, we study intelligent ways of integrating recurrent block in common supervised depth estimation pipeline. We propose a novel method, which takes advantage of the convolutional gated recurrent unit (convGRU) and convolutional long short-term memory (convLSTM). We compare use of convGRU and convLSTM blocks and determine the best model for real-time depth estimation task. We carefully study training strategy and provide new deep neural networks architectures for the task of depth estimation from monocular video using information from past frames based on attention mechanism. We demonstrate the efficiency of exploiting temporal information by comparing our best recurrent method with existing image-based and video-based solutions for monocular depth reconstruction.

## Introduction

Recently, advances in deep learning and computer vision have greatly influenced such rapidly growing fields as robotics, augmented reality and self-driving cars. A major progress have been made in depth estimation field playing important role in safety and vision systems. Originally stated as a supervised learning problem of depth estimation from RGB images, a lot of improvements were presented over the past five years, which boosted depth prediction accuracy (Saxena, Sun & Ng, 2007; Eigen, Puhrsch & Fergus, 2014; Eigen & Fergus, 2015; Laina et al., 2016; Fu et al., 2018). Recently, self-supervised depth estimation methods, which rely on the camera motion, have also been improved (Zou, Luo & Huang, 2018; Godard et al., 2019; Zhou et al., 2017; Yin & Shi, 2018). In addition, depth completion based on sparse depth information, produced by a LiDAR sensor or SLAM, has also improved its robustness and accuracy of depth estimation (Mal & Karaman, 2018; Ma, Cavalheiro & Karaman, 2018; Uhrig et al., 2017; Van Gansbeke et al., 2019).

Despite the huge progress in all three directions, it seems that there is still room to grow. In particular, it is necessary to mention some of the disadvantages of three settings. The depth completion setting is sensitive to lighting conditions, the self-supervised depth estimation setting does not rely on ground truth depth and partly utilize the ego-motion information but still has not beat the state-of-the-art supervised methods; the supervised depth estimation from RGB images is highly dependant on accurate ground truth, but at the same time is an affordable solution considering the costs.

Current depth estimation methods, based on using just a single image, are inherently ambiguous and unreliable. These methods are not robust enough and are sensitive to noise. In order to make this methods more precise, it is necessary to provide new ideas of exploiting additional information to make predictions applicable for the monocular vision setting.

If we consider the mobile robot applications, which perceive the world as a video stream, the necessity for the method, which could utilize the temporal dependency across frames, is very high. Looking at the self-supervised methods, one can see, that they already use video in the training stage for computing the view-synthesis loss across nearby frames (Godard et al., 2019; Zhou et al., 2017), but they still do not utilize temporal information at testing stage. Recently, several works were conducted on unsupervised video depth estimation methods, which removed the need for supervised information (Mahjourian, Wicke & Angelova, 2018; Wang et al., 2018).

Considering the sequence-to-sequence tasks, an attention mechanism was proposed in Bahdanau, Cho & Bengio (2014) which evolved in transformer architectures showing state-of-the-art results in the tasks such as machine translation (Vaswani et al., 2017). Back to the video depth estimation, these methods work with frame sequences; hence, it is interesting whether and how much integration of the attention mechanism in the recurrent-based pipeline can improve depth estimation.

In this work, we first implement a supervised depth estimation method and then consider it as a baseline for next experiments, which include architecture modifications and different training strategies. Further, we integrate the encoder–decoder network with recurrent block, which can be a convolutional LSTM (convLSTM) and a convolutional GRU (convGRU). The usage of the recurrent block increases the accuracy of depth estimation by leveraging the temporal information across frames. We propose a novel architecture integrated with attention mechanism, which outperforms current best supervised recurrent depth estimation methods. We report results of our study on the KITTI Dataset (Geiger et al., 2013), which contains outdoor depth and RGB data. Our method trains and tests on time series of data.

**Figure 1:**
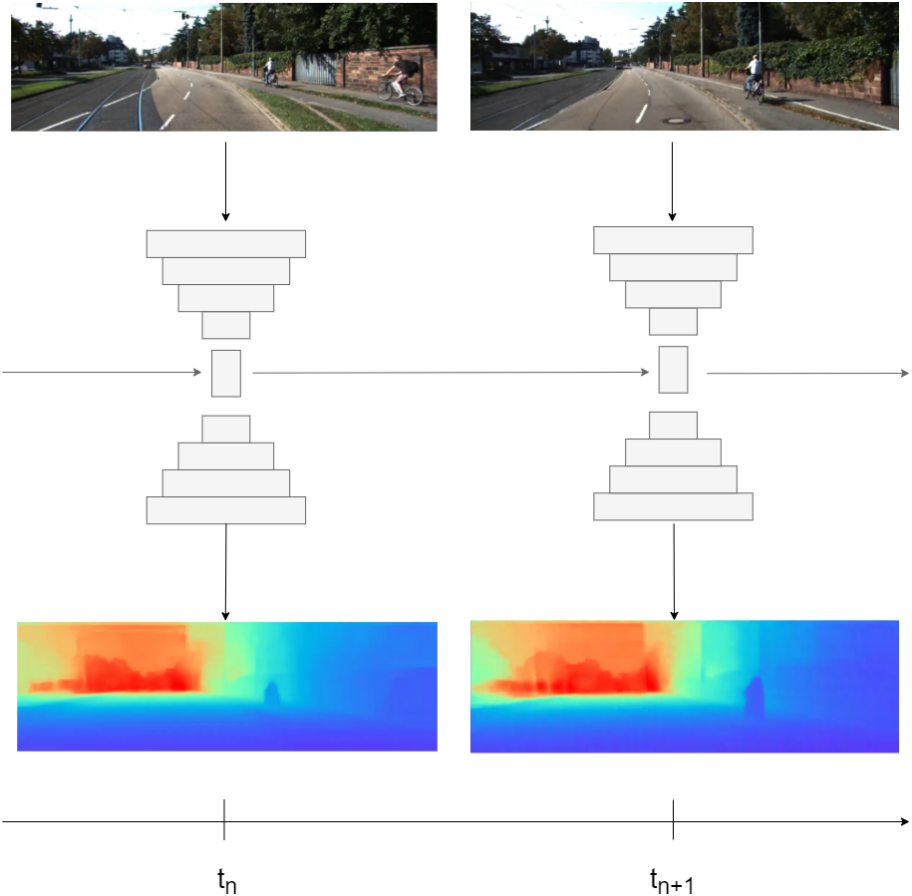
Illustration of our method. First, image is passed through encoder, then encoder bottleneck features are passed to recurrent block. At final, in order to get depth prediction, the hidden state is passed to decoder. Road images and ground truth depth maps credit: Geiger et al. (2013).

To summarize, our main contributions are as follows:

1. We propose a novel recurrent network, integrated with attention mechanism, which takes advantage of convGRU or convLSTM to leverage temporal information in the depth estimation task;
2. We design an effective training strategy for the recurrent-based solutions in dense depth prediction tasks as shown in Fig. 1;
3. We provide a result of extensive experiments and ablation studies. These experiments show that our recurrent method based on convGRU or convLSTM outperforms the current state-of-the-art methods.

## Related works

In this section, we first overview supervised depth estimation methods. Next, we discuss advances in the self-supervised based approaches. Finally, we consider the video estimation methods which are of great importance for our work.

### Supervised depth estimation

Depth estimation from a single image is classified as an ill-posed problem, as long as one input image can be projected to multiple plausible depths (across sensors and methods of acquisition). Recently, many depth estimation methods, which are based on the deep learning, have achieved great results.

In Xie, Girshick & Farhadi (2016), shortcut connections were used in network in order to fuse low-level and high-level features. In Eigen, Puhrsch & Fergus (2014), authors used a multi-scale neural network with two components in order to generate coarse estimations globally and refine the corresponding results locally. Another view on the depth estimation problem was introduced in Cao, Wu & Shen (2018), where authors formulated a classification problem instead of a regression problem.

Further improvements required new ideas for loss functions. In Laina et al. (2016), authors employed a reverse Huber loss to estimate depth distributions, while in Yin et al. (2019) authors implemented the loss term that enforces geometric constraints. Same time, perceptual loss, which was introduced in Johnson, Alahi & Fei-Fei (2016), was successfully used in the dense depth estimation task (Wang et al., 2018a; Makarov et al., 2017). To further boost performance, some works have integrated continuous CRFs in their DL framework (Liu et al., 2015).

One of the ways to enhance performance of the methods, which predict depth from RGB images, is to use additional information from other sources. One such example of a source is sparse depth, which could be extracted from SLAM systems (Mal & Karaman, 2018). A LiDAR sensor can also serve as a sparse depth input (Liao et al., 2017). Model based on the semi-dense depth interpolation was presented in Makarov, Aliev & Gerasimova (2017), where authors proposed an end-to-end learnable residual convolutional neural network architecture, that achieved fast interpolation of semi-dense depth maps. The suggested approach was later improved for fast depth estimation from sparse (Makarov, Korinevskaya & Aliev, 2018a; Makarov, Korinevskaya & Aliev, 2018b) and low resolution (Korinevskaya & Makarov, 2018) depth values.

Recently, excellent performance was achieved in Fu et al. (2018), where authors proposed a SID policy and ordinal regression loss. Although, the results are great, this method can not be used in the mobile robotics platforms, due to complex network architecture, thus, it can not be applied in real-time for video processing.

In general, the problem of encoder–decoder architectures or GANs for the supervised depth estimation lies either in current limitations for error improvement or slow performance making approaches inapplicable for real-world scenarios on constraint resources.

### Self-supervised depth estimation

Acquiring the ground truth depth is quite a challenging task. That is why the alternative methods rise, where image reconstruction is used as a supervisory signal. Mainly, there are two types of methods: one use stereo pairs for the training, while another use monocular sequences. Thus, this type of models are trained via minimizing the image reconstruction error, where depth for certain image is projected in nearby views.

For the methods, which use stereo pairs as input, the pixel disparities between the synchronized stereo pair are predicted during training. Authors from (Xie, Girshick & Farhadi, 2016) introduced a model with discretized depth in context of the novel view synthesis problem. In Godard, Mac Aodha & Brostow (2017), authors enhanced performance by using left–right view consistency as the supervisory signal, while in Garg et al. (2016) performance was improved by predicting continuous disparity values.

Using monocular sequences as input leads to slightly different methods, as long as camera pose estimation between frames plays a crucial role in the whole training pipeline. Estimation of the camera pose is quite challenging, due to the object motion, but it only requires during training stage. Great progress has been made in this field, starting from Zhou et al. (2017), where separate pose network was trained along depth estimation network, to Godard, Mac Aodha & Brostow (2017), where a novel multi-scale sample method and an auto-masking loss were introduced.

Although the sequential video frames are used in view-synthesis loss, the spatiotemporal data at longer range is still missing.

### Video depth estimation

One of the earliest work in this field was Karsch, Liu & Kang (2014), in which authors improved depth estimation by using local motion cues and secured temporal depth consistency via optical flow. However, this method is offline, which is not suitable for our online setting. In Ranftl et al. (2016), consecutive frame information was used for optical flow segmentation and depth estimation via geometry reconstruction. In Xu et al. (2017), depth estimation based on multi-scale convolutional neural networks with continuous Conditional Random Fields (CRFs) refinement was proposed. The authors used either cascade of multiple CRFs, or unified graphical model. Later, several works were presented, which focused on unsupervised video depth estimation methods (Mahjourian, Wicke & Angelova, 2018; Wang et al., 2018b).

Recently, convLSTM was proposed for the weather forecasting task (Xingjian et al., 2015) and convGRU was introduced for solving both human action recognition and video captioning tasks (Ballas et al., 2015). Recently, convLSTM was successfully used in the real-time video depth estimation task (Zhang et al., 2019), in which authors boosted performance with temporal consistency loss and generative adversarial learning scheme. In Vaishakh et al. (2020), authors have also exploited convLSTM in the real-time depth estimation task, however, they focused mostly on self-supervised setting and training strategy, which involved pre-training of the initial hidden states.

Thus, according to our knowledge, still there were no works, which integrated the convGRU block in real-time depth estimation pipeline for videos and, hence, there were no comparison between convGRU and convLSTM in this online setting. As we know, GRU is yet another gated architecture. In Chung et al. (2014), authors showed, that GRU has similar performance in comparison with LSTM. Also, reduced number of gates leads to fewer parameters in model, which reduces complexity of the whole pipeline and that is a crucial point for online mobile robot applications. That is why we compare both recurrent blocks: convGRU and convLSTM as a part of the real-time depth estimation task.

Integration of the recurrent block in pipeline already leads to depth estimation accuracy improvement. Moreover, this pipeline can be further enhanced by explicitly exploiting previous frames information, particularly by using attention mechanism as we show below.

## Method

In this section, we first summarise the supervised depth prediction pipeline, which will be used as a baseline in our work. Then we describe two recurrent blocks: convLSTM and convGRU. Next, we describe the common recurrent depth estimation pipeline and role of convLSTM and convGRU in it. In the end of this section, we provide description of possible architecture modifications, which explicitly exploit previous frames information. Particularly, we propose the attention based modification, which aggregates information from previous frames.

Here we also define certain notations to simplify description of the following methods. Let us denote *X* ∈ *R*^*m*×*n*^ as input RGB image, *Y* ∈ *R*^*m*×*n*^ as ground truth depth, where *n* denotes height and *m* denotes width. Since we work with sequences, it is also important to denote by *t* a timestamp of data time series 1, 2, 3..., *T*, where T equals the length of frame sequence. The image and depth data are considered to be synchronized and for timestamp *t* denotes as *X*_*t*_ and *Y*_*t*_, respectively.

### Supervised depth estimation

We formulate the depth estimation task as a regression problem: The task is to learn function *f*:*X*→^*Y*^, where *X* is an input RGB image and $\hat {Y}$ is a predicted depth, where $\hat {Y}$ minimizes loss function $L(\hat {Y},Y)$, where *Y* correspond to ground truth depth. We follow the common architecture for the depth estimation network and represent it as encoder–decoder (following notations of Vaishakh et al. (2020)). Thus, it takes the next form:

(1) \begin{eqnarray*}Z={f}_{enc}(X),\quad \hat {Y}={f}_{dec}(Z),\end{eqnarray*}

where *Z* correspond to encoder bottleneck features.

Another subject to consider is the choice of a loss function. The common loss functions are: *L*_1_, *L*_2_ and Reversed Huber loss (denoted as berHu), which was introduced in Owen (2007). According to Makarov, Aliev & Gerasimova (2017), *L*_2_ loss function tends to give oversmoothed results and often has poor perceptual quality. Same time, Mal & Karaman (2018) showed that using *L*_1_ loss leads to better results compared with berHu loss. Following mentioned above considerations, we decided to use *L*_1_ loss in our work. Following (Mal & Karaman, 2018), we apply a binary mask *M*_*i*,*j*_ of dimensions *m* × *n*, where *M*_*i*,*j*_ = 1 for valid values of the ground truth depth map $\hat {Y}$. Same as in Godard, Mac Aodha & Brostow (2017), we add an edge-aware smoothing loss term, which can be introduced as:

(2) \begin{eqnarray*}{L}_{smooth}={|}{\partial }_{x}{\hat {Y}}^{\ast }{|}{e}^{{|}{|}-{\partial }_{x}X{|}{|}}+{|}{\partial }_{y}{\hat {Y}}^{\ast }{|}{e}^{{|}{|}-{\partial }_{y}X{|}{|}},\end{eqnarray*}

where ${\hat {Y}}^{\ast }=\hat {Y}/\hat {\bar {Y}}$ is mean normalized inverse depth from Wang et al. (2018b) to prevent shrinking of the estimated depth.

In the next section we describe two recurrent blocks: convGRU and convLSTM, which will be integrated between encoder and decoder modules.

### Recurrent blocks: convLSTM and convGRU

Recently, LSTMs achieved great results in various sequence-to-sequence tasks, for example in speech recognition (Graves, rahman Mohamed & Hinton, 2013) and machine translation (Parmar & Devi, 2018). Long and short term temporal dependencies can be captured by utilizing the memory cell mechanism. Still, standard LSTM uses one dimensional input, that is why we can not directly apply it to image sequences. Xingjian et al. (2015) overcame this problem by introducing the convLSTM mechanism, which allowed to handle two-dimensional feature maps. In this case *i*_*t*_, *f*_*t*_, *o*_*t*_ gates, cell outputs *C*_*t*_, and hidden states *H*_*t*_ are 3D tensors, which last two dimensions are spatial dimensions.

The original structure of the convLSTM cell did not work properly in our experiments, that is why we used the cell structure, described in Zhang et al. (2019). The key equations are shown below in ??, where ∗ denotes convolutional operator and *W*_*xk*_, *W*_*hk*_, and *b*_*k*_, *k* ∈ {*f*, *i*, *o*, *c*}, denotes the kernel and bias terms for the corresponding convolutional layers:

(3) \begin{eqnarray*}{f}_{t}=\sigma ({W}_{xf}\ast {X}_{t}+{W}_{hf}\ast {H}_{t-1}+{b}_{f}),\nonumber\\\displaystyle {i}_{t}=\sigma ({W}_{xi}\ast {X}_{t}+{W}_{hi}\ast {H}_{t-1}+{b}_{i}),\nonumber\\\displaystyle {o}_{t}=\sigma ({W}_{xo}\ast {X}_{t}+{W}_{ho}\ast {H}_{t-1}+{b}_{o}),\tilde {{C}_{t}}=tanh({W}_{xc}\ast {X}_{t}+{W}_{hc}\ast {H}_{t-1}+{b}_{c}),\nonumber\\\displaystyle {C}_{t}={f}_{t}\circ {C}_{t-1}{i}_{t}\circ \tilde {{C}_{t}},\nonumber\\\displaystyle {H}_{t}={o}_{t}\circ tanh({C}_{t})\end{eqnarray*}

GRU follows the same gated principal as LSTM, but with a little simpler architecture. It has reduced number of gates thus fewer parameters (Chung et al., 2014). The ConvGRU was first introduced in Ballas et al. (2015) for the video captioning task. It also performed well in the video segmentation task, as shown in Siam et al. (2017). Equation (4) describe mathematical model of the ConvGRU, where * is a convolutional operator, *z*_*t*_, *r*_*t*_ correspond to gates, *H*_*t*_ correspond to a hidden state, *W*_*xk*_, *W*_*hk*_, and *b*_*k*_, *k* ∈ {*z*, *r*, *h*} correspond to kernel and bias terms, respectively:

(4) \begin{eqnarray*}{z}_{t}=\sigma ({W}_{xz}\ast {X}_{t}+{W}_{hz}\ast {H}_{t-1}+{b}_{z}),{r}_{t}=\sigma ({W}_{xr}\ast {X}_{t}+{W}_{hr}\ast {H}_{t-1}+{b}_{r}),\tilde {{h}_{t}}=tanh({W}_{x}\ast {X}_{t}+{W}_{h}\ast ({r}_{t}\circ {H}_{t-1})+{b}_{h}),{H}_{t}=(1-{z}_{t})(\circ {H}_{t-1}+z\circ \tilde {{h}_{t}})\end{eqnarray*}

**Figure 2:**
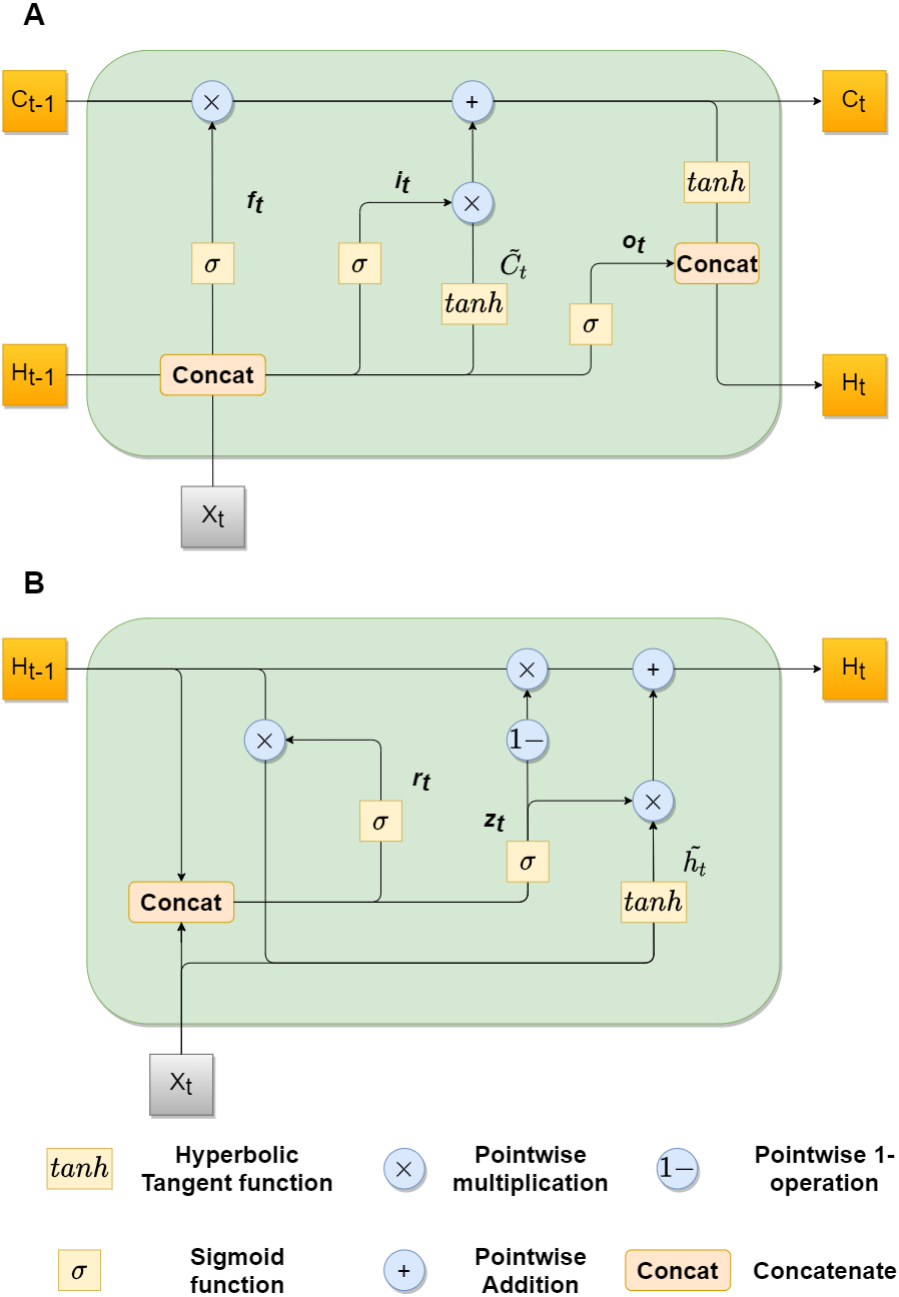
convLSTM (A) and convGRU (B) blocks with feature maps as inputs.

Figure 2 shows architectures of both convLSTM (A) and convGRU blocks (B).

In the next section, we will declare the recurrent depth estimation pipeline and demonstrate the role of recurrent blocks described above in it.

### Supervised recurrent depth prediction

In ‘Supervised depth estimation’ we described a baseline encoder–decoder architecture, which estimates depth for a single frame separately from other frames, which leads to loss of the spatiotemporal information across sequence. In this section we formulate the recurrent depth estimation problem and formalize the convGRU and the convLSTM role in recurrent pipeline.

The problem is formulated as follows: at timestamp *t*, given the encoder representation *Z*_*t*_ for image *X*_*t*_ and given previous hidden states of the recurrent block (*H*_*t*−1_, *H*_*t*−2_, .., *H*_*t*−*n*_), we need to estimate depth map $\hat {{Y}_{t}}$:

(5) \begin{eqnarray*}\hat {{Y}_{t}}=\argmin _{\tilde {{Y}_{t}}}P(\tilde {{Y}_{t}}{|}{Z}_{t},{H}_{t-1},{H}_{t-2},..,{H}_{t-n}),\end{eqnarray*}

where *n* equals to the number of frames from the beginning of the sequence.

So, after adding the recurrent block, which can be either a convGRU or a convLSTM, the basic supervised depth estimation pipeline looks like this: at time *t*, we pass image *X*_*t*_ through the encoder *Z*_*t*_ = *f*_*enc*_(*X*_*t*_), then we pass *Z*_*t*_ through convLSTM

(6) \begin{eqnarray*}{H}_{t},{C}_{t}={f}_{convLSTM}({Z}_{t},{H}_{t-1},{C}_{t-1}),\end{eqnarray*}

or through convGRU

**Figure 3:**
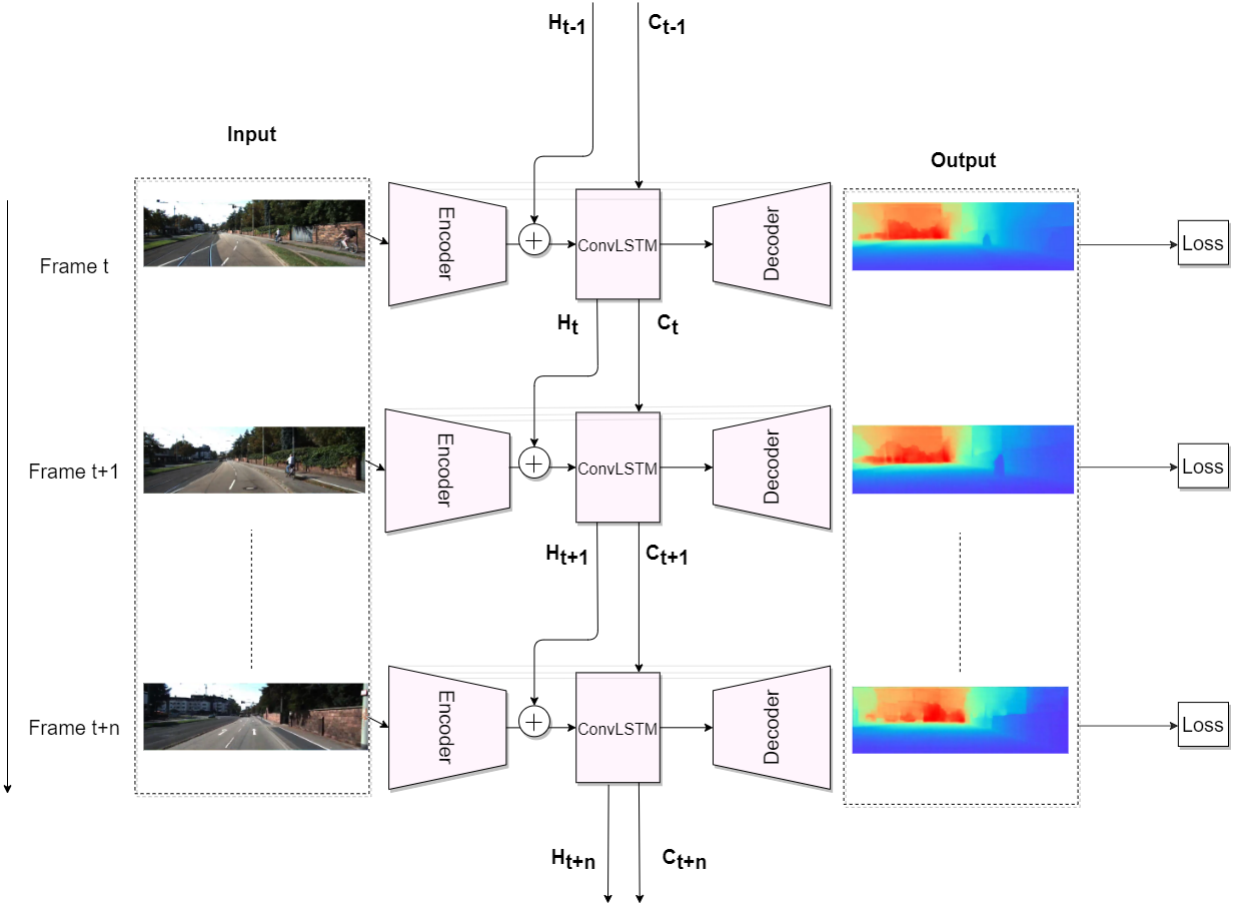
Recurrent Depth Estimation Pipeline with ConvLSTM as recurrent block. Road images and ground truth depth maps credit: Geiger et al. (2013).

(7) \begin{eqnarray*}{H}_{t}={f}_{convGRU}({Z}_{t},{H}_{t-1}),\end{eqnarray*}

where *H*_*t*_ and *C*_*t*_ correspond to hidden and cell states, respectively. Finally, in order to get a depth estimation ${\hat {Y}}_{t}$, a hidden representation *H*_*t*_ is passed through decoder ${\hat {Y}}_{t}={f}_{dec}({H}_{t})$. Figure 3 demonstrates the basic recurrent depth estimation pipeline with convLSTM as recurrent block.

Thus, the integration of recurrent block in our pipeline helps us to capture and exploit the spatiotemporal information across frame sequence. In our work, we get boost in the performance, in comparison with the non-recurrent approach. We explain it by ability of the recurrent block to capture motions of visual components via transitional kernels, whereas we consider a hidden state as a hidden representation of visual structure according to Xingjian et al. (2015).

Integration of the recurrent block in the non-recurrent based architecture, already gives us improvements in the depth prediction accuracy. However, there are still ways to improve current architecture, by exploiting previous frames information for current frame depth prediction. In the next section, we propose yet simple, but quite efficient architecture modifications.

### Exploiting previous frames information

Recently, there were few works, which focused on exploiting the temporal information via concatenation of multiple frames at input, such as Sun et al. (2015) and Diba et al. (2019). The problem of these approaches lies in inability to scale well on long sequences. Same time, the explicit injection of previous frame information can benefit in predicting the depth for current frame was suggested in Kaneko & Yamamoto (2017). Thus, in this section, we propose three modifications to the recurrent depth estimation pipeline, which utilize previous frames information.

One of straightforward methods is the explicit injection of just one previous frame, which can be described as follows:

(8) \begin{eqnarray*}\hat {{Y}_{t}}={f}_{dec}(g({H}_{t},{H}_{t-1})),\end{eqnarray*}

where function *g* refers to either concatenation or fusion. Although, the explicit injection can be useful, it can not give significant boost in the performance, due to a little difference between adjacent frames. Hence, we propose a third attention-based modification, which aggregates information from previous *k* frames hidden representations.

First, let us denote *f*_*RecBlock*1_, *f*_*RecBlock*2_ as Layer 1 and Layer 2 of the recurrent block respectively, where recurrent block can be either a convGRU or a convLSTM. Let us refer to *H*1_*t*_, *H*1_*t*−1_, …, *H*1_*t*−*k*_ and *H*2_*t*_, *H*2_*t*−1_, …, *H*2_*t*−*k*_ as hidden states at timestamps *t*, *t* − 1, …, *t* − *k* for the Layer 1 and the Layer 2 respectively. The idea is to form the context vector, which preserve information from k previous frames. We propose an attention mechanism, which is based on relevance between two hidden states.

Let us define an alignment score between hidden state *s* and *v* as:

(9) \begin{eqnarray*}score({H}_{s},{H}_{v})={H}_{s}^{T}{H}_{v},\end{eqnarray*}

which is a scalar product between two vectors. The alignment weights for the previous k frames are obtained by the following formula:

(10) \begin{eqnarray*}{\alpha }_{t}(s)= \frac{score(H{1}_{t},H{1}_{t-s})}{\sum _{{s}=1}^{k}score(H{1}_{t},H{1}_{t-{s}})} ,\end{eqnarray*}

where *s* ∈ 1, 2.., *k*.

To get the context vector for the current timestamp *t*, we do weighted average on k previous hidden states from Layer 2:

(11) \begin{eqnarray*}{u}_{t}=\sum _{s=1}^{k}{\alpha }_{t}(s)H{2}_{t-s}\end{eqnarray*}

Thus, we obtain attention scores from Layer 1 hidden states and construct context vector from Layer 2 hidden states. For the final step, we concatenate the context vector and the hidden state for timestamp t and pass it to decoder:

(12) \begin{eqnarray*}\hat {{Y}_{t}}={f}_{dec}(H{2}_{t}\oplus {u}_{t}),\end{eqnarray*}

**Figure 4:**
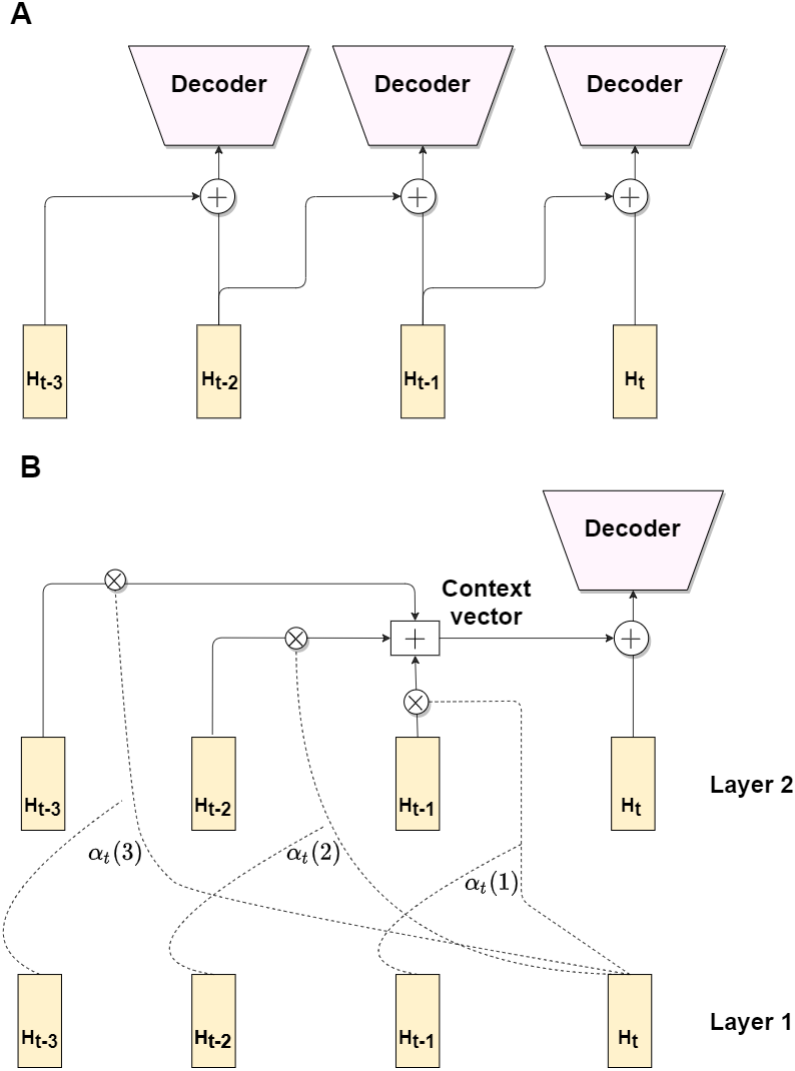
Architecture modifications. Modifications 1 and 2 exploit previous frame hidden state via concatenation/fusion with current frame hidden state (A). Modification 3 utilize attention mechanism (B).

Figure 4 contains illustrations for all three modifications. While first two modifications (A) are rather simple and can not give significant enhance in depth prediction accuracy, due to exploiting information only from the previous frame, the third one (B) is more complex and provide an accuracy improvement, by utilizing attention-based mechanism. In section ‘Experiment with model modifications’ we analyze the benefits of the proposed modifications in comparison with baseline non-recurrent and recurrent pipelines.

It is important to note few things:

• Before constructing the context vector, all hidden representations are reshaped from 3-dimensional to 1 dimensional vector;
• Due to high dimensionality of hidden states, it is impossible to implement the classical softmax attention mechanism, as long as it contains exponent step.

## Training Framework

In this section we first describe the data used in the course of work. Then we provide the training details and the baseline architecture implementation details. Then we discuss the training strategy and provide system configuration details.

### Dataset

In our work, we report results on KITTI Dataset (Geiger et al., 2013). This dataset contains 61 outdoor video scenes captured by cameras and depth sensors, fixed on a driving car. The initial resolution of the videos is 375x1242. Training on frame sequences is quite challenging task, considering the GPU RAM issues. Hence, in order to decrease memory costs, in all our experiments we resize images to a resolution of 192 × 640. Works Vaishakh et al. (2020) and Godard et al. (2019) use the same resolution, which leads to fair comparison with their results. We use Eigen split, defined in Eigen & Fergus (2015), which separates 32 monocular sequences for training and 29 sequences for testing. 697 specific samples from test sequences are used as a standard evaluation test set (Vaishakh et al., 2020; Godard et al., 2019) for the non-recurrent based experiments. Considering the recurrent-based experiments, we split 32 sequences into 27 for training and 5 for validation. We divide sequences into small sub-sequences of 10 frames long. This sub-sequences are used as training samples. During test phase we evaluate on complete video sequences. We cap the maximum predictions to 80 m.

### Training Details

For non-recurrent based experiments we use the batch size of 24; while for recurrent based experiments we use the batch size of 6, due to GPU RAM limitations, as long as we use sequences as training samples. We resize all images to a resolution of 192 × 640, due to GPU RAM limitations. We choose the Adam optimizer and the learning rate of 10^−4^. We apply exponential decay for learning rate with the decay rate 0.96. For the smooth loss term, we use weight 0.001. To achieve competitive results, we follow Vaishakh et al. (2020), Godard et al. (2019) and Luo et al. (2018) and use pretrained ImageNet (Krizhevsky, Sutskever & Hinton, 2017) weights for the encoder. Additionally, we apply following augmentations:

• color and depths are both horizontally flipped with a 50% chance;
• brightness, contrast, saturation and hue jitter with respective ranges of ±0.2, ±0.2, ±0.2 and ±0.1.

For the attention modification, we look at previous 3 frames. We train both recurrent-based and non-recurrent based architectures for 20 epochs.

### Baseline architecture

For the baseline approach, we follow the U-net work (Ronneberger, Fischer & Brox, 2015) and use the encoder–decoder architecture with skip connections, where ResNet-18 (He et al., 2016) performs the role of an encoder. As for the decoder, we apply upconvolutional blocks same as in DispNet work by Mayer et al. (2016). At the final step, we get the depth prediction via inverse transformation of disparity output: $\hat {Y}=1/(a\sigma +b)$, where *a* and *b* are selected to limit depth $\hat {Y}$ from 0.1 to 100 m.

The basic approach is expanded to recurrent, by adding a recurrent module between the encoder and decoder, either convGRU presented in Ballas et al. (2015) or convLSTM preented in Xingjian et al. (2015), such that encoder output is passed to this module. Details are provided in ‘Supervised recurrent depth prediction’.

### Training strategy

There are several important aspects to consider when we design the training strategy for the recurrent depth estimation pipeline.

The first one is about the impact of the initial hidden state of a recurrent block. As we know, in a vanilla LSTM and in a vanilla GRU, the latter are initialized by zeros. This is a common practice, taking into account the tasks in which those blocks are applied (for example, Machine Translation or Time Series Analysis). It works fine in these type of tasks for several reasons: length of sequences is relatively long compared to the size of hidden state and the impact of initial hidden state is trivial. However, when we deal with monocular videos, we have some restrictions on memory resources, which leads to shortening the length of sequences at training stage. Thus, the impact of the initial hidden state becomes crucial. Following work (Vaishakh et al., 2020), we divide our training process in two stages: at the first stage, we consider the initial hidden state as learnable parameter, then, at the second stage, the trained initial hidden state is used at the start of every frame sequence.

The second aspect concerns the impact of the pretrained encoder and the pretrained decoder on the training process of the recurrent pipeline. As we know, the common way is to train architecture in end-to-end manner, however, we can let the recurrent block to adapt to sequence faster, by preloading weights, which were obtained as a result of training in the supervised baseline approach. Thus, we perform a finetuning of architecture with additional recurrent block on monocular video sequences.

### System configuration

All models were implemented with Pytorch Paszke et al., 2017. All experiments were carried out with the RTX6000 GPU, Intel(R) Xeon(R) CPU and 32 Gb RAM. The operating system is Ubuntu 18.04. All process was performed using free and open-source distribution Anaconda with python version 3.7.4.

## Experiments

In this section we analyze results of conducted experiments and compare our best result with current state-of-the-art methods.

### Experiment with model modifications

The experiments are carried out in the following order: At first, we train a non-recurrent architecture (Table 1 Baseline row). Then we add a recurrent block between encoder and decoder modules and train in end-to-end manner. We test both convGRU and convLSTM (Table 1 convGRU and convLSTM rows). After that, we preload encoder and decoder weights, received from the first experiment, and train in same manner (Table 1 recurrent Block + weights rows). Then we test all the architecture modifications: concatenation/fusion of previous frame hidden state with current frame hidden state and modification based on attention mechanism. Following (Vaishakh et al., 2020), we experiment with activation function: Tanh activation function is replaced with ELU activation function (Table 1 last row). We conduct this change only with convGRU architecture, because activation function change in convLSTM cell leads to worse results.

**Table 1:** Results for supervised depth prediction in conducted experiments. Bold corresponds to the best metric.

Method	↓ RMSE
Baseline	4.397
convLSTM	4.536
convLSTM + weights	4.181
convLSTM + weights + mod. 1	4.203
convLSTM + weights + mod. 2	4.205
convLSTM + weights + mod. 3	4.168
convGRU	4.437
convGRU + weights	4.188
convGRU + weights + mod. 1	4.210
convGRU + weights + mod. 2	4.211
convGRU + weights + mod. 3	4.196
convGRU + weights + mod. 3 + ELU	**4.104**

The results of these experiments are presented in Table 1. As we can see, the recurrent-based architectures, which use preloaded weights from the non-recurrent based model, outperforms the baseline supervised model (4.181 against 4.397 RMSE). Moreover, we see an improvement by 8% in comparison with the model, trained from scratch, both for convLSTM, convGRU, which confirms the correct use of preloaded weights for encoder, decoder blocks. As we can see, convLSTM slightly outperforms convGRU (by 0.007 RMSE), however the difference is not that great. By using the attention modification, the results are improved even further. First two modifications, which utilize previous frame information by doing concatenation/fusion with current frame hidden state, only make results worse. This happens due to little difference between adjacent frames.

It is important to note, that the attention modification works better with convLSTM (4.168 RMSE). At last, the change of Tanh activation function to ELU activation function in convGRU cell, leads to even better results (4.104 RMSE). We see an improvement, comparing with Tanh activation function, because, in this case we match the scale with the output of encoder. Summing up, the attention-based recurrent architecture showed an improvement in comparison with baseline non-recurrent architecture by 0.293 RMSE, which demonstrates the effectiveness of proposed method.

### Comparison with state-of-the-art models

Table 2 contains results from previous state-of-the-art works and result of our best recurrent-based architecture. By comparing results of our best supervised-based approach with other supervised approaches, we see a significant improvement. Fu et al. (2018) method still outperforms our method, however, their method is not suitable for real-time depth estimation task (autonomous driving), due to complex network architecture; also it provides poorer results in terms of visual perception compared to our approach. The results of speed tests on Nvidia GTX-1060 with MAX-Q Design GPU are shown in Table 3.

**Table 2:** Comparison with state-of-the-art methods. First column classifies methods as ‘s’ (supervised), ‘u’ (self-supervised/unsupervised), ‘v’ (video based).

	Method	↓ RMSE	↓ RMSE(log)	↓ Abs Rel Diff	↓ Sq Rel Diff	↑*δ* < 1.25	↑*δ* < 1.25^2^	↑*δ* < 1.25^3^
s	Eigen, Puhrsch & Fergus (2014)	7.156	0.270	0.215	1.515	0.692	0.899	0.967
s	Wang, Pizer & Frahm (2019)	5.106	0.211	0.128	0.908	0.647	0.882	0.961
s	Kuznietsov, Stckler & Leibe (2017)	4.621	0.189	0.113	0.741	0.862	0.960	0.986
s	Guo et al. (2018)	4.422	0.183	0.105	0.717	0.874	0.959	0.983
s	Yang et al. (2018)	4.442	0.187	0.097	0.734	0.888	0.958	0.980
s	Liu et al. (2015)	6.986	0.289	0.217	1.841	0.647	0.882	0.961
s	Fu et al. (2018)	**2.727**	**0.120**	**0.072**	**0.307**	**0.932**	**0.984**	**0.994**
u	Casser et al. (2018)	4.750	0.187	0.109	0.825	0.874	0.958	0.982
u	Godard et al. (2019)	4.863	0.193	0.115	0.903	0.877	0.959	0.981
v	Zhang et al. (2019)	4.137	–	0.101	–	0.890	0.970	0.989
v	Vaishakh et al. (2020)	4.148	0.172	0.102	0.655	0.884	0.966	0.987
v	The method in this study	**4.104**	**0.170**	**0.101**	**0.707**	**0.887**	**0.964**	**0.986**

**Notes.**

Best results in real-time and not real-time are marked as bold and as bold & underlined, respectively.

**Table 3:** Processing speed of different methods measure in rames per second (fps).

Approach	Time (ms per frame)	Speed (fps)
Baseline	14.2 ± 0.9	70 ± 4
convGRU	15.7 ± 0.8	64 ± 4
convGRU + Attention	17.4 ± 0.8	58 ± 4
convLSTM	15.9 ± 0.7	64 ± 4
convLSTM + Attention	17.6 ± 0.6	57 ± 4
DORN (Fu et al., 2018)	69.2 ± 0.6	15 ± 3

Our method, based on convGRU and attention mechanism, shows better results, than methods, based on convLSTM in Zhang et al. (2019) and Vaishakh et al. (2020), which proves efficiency of attention-based modification together with online performance.

**Figure 5:**
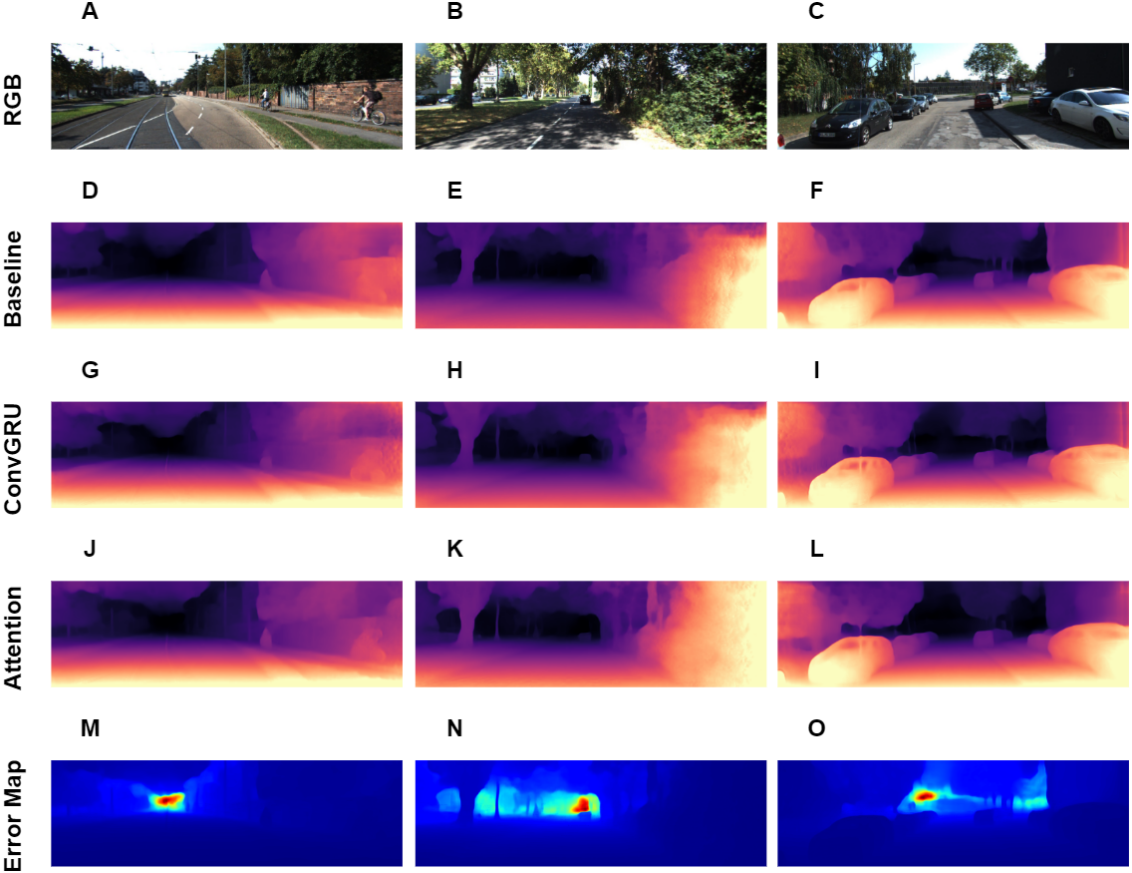
Visual results of depth estimation for different approaches on KITTI dataset. A, B, C correspond to RGB images. D, E, F correspond to depth output, produced by Baseline model. G, H, I correspond to depth output, produced by model with ConvGRU block. J, K, L correspond to depth output, produced by model with ConvGRU and attention blocks. M, N, O correspond to error maps. Brighter colors on error maps mean higher errors. Road images and ground truth depth maps credit: Geiger et al. (2013).

## Discussion

Attention-based module improves quality over straightforward recurrent based approach (convGRU / convLSTM). The explicit injection of past frame information directly benefits to depth estimation, for example, in cases, when self-driving car makes a turn on the street and focusing on past few frames (forming the attention-based context vector) helps to improve depth prediction on certain areas of image. When we are working with feature map sequences, we can not apply GRU/LSTM/Attention in a straightforward way. Previously, authors used attention in works, focused on the depth estimation. For example, in Xu et al. (2018), authors implemented a structured attention model which automatically regulates the amount of information transferred between corresponding features at different scales, and in Chen, Zhao & Hu (2019), authors integrated the self-attention module to mitigate grid artifacts. Yet, there were no works on the video depth estimation, which focused on integrating the attention mechanism with the recurrent block (convGRU/convLSTM).

In our architecture, we utilize an attention mechanism, applied to feature maps sequences, and it is the first article to provide solid proof of successful SOTA outperformance for depth estimation in online setting using attention-based mechanism. Unfortunately, comparison with Vaishakh et al. (2020) is not possible due to irreproducible results from the paper, following which we obtained much worse results compared to the reported in the paper (recurrent depth estimation pipeline with convolutional LSTM). On the contrary, we provide reproducible results with rigorously extended approaches and aim to publish code accompanying paper.

We used one dataset (KITTI) to match the evaluation benchmarks, mentioned in other papers. To provide fair comparison results, we follow a unified approach of train/test split and evaluation presented in studies on video-depth estimation.

Overall, we present a novel architecture for the task, which outperforms other approaches in *online depth estimation* setting. The visual results are presented in Fig. 5, it also contains error maps for our best method. From the error maps we can see, that error is large in the far areas with high depth variance.

## Conclusion and Future work

In this work we introduced a novel method for estimating time-series of dense depth maps, based on convGRU module and attention mechanism. A recurrent framework has been developed for supervised depth prediction task. Our method demonstrates improvement on KITTI dataset, in comparison with other state-of-the-art methods. Our framework is able to execute in real-time for mobile robot applications.

An interesting direction of future work will be to adapt our current framework for self-supervised depth prediction task. The recurrent pipeline can strongly benefit self-supervised learning, which already uses idea of using information from video to provide temporally coherent depth predictions.

It is also interesting to mention that unsupervised and self-supervised frameworks may achieve great performance while being combined with segmentation and pose estimation guidance. In simple words, if you have robust self-supervised model and a place in image, in which you can reconstruct ground truth depth, then you can reconstruct dense depth map with high precision leading to much less efforts on high-precision depth sensors or necessity to label data for various environment conditions. To find such places, different SLAM algorithms and anchor-less detectors may be of great use.

Another interesting area of further research direction is to investigate the performance of our framework in indoor environment. The framework may benefit from smaller variance of depth values and lead to better performance for indoor stable camera movement.

Finally, as mentioned by one of the reviewers, using Lidars providing high precision low-res depth map may benefit depth completion problem. The problem is that Lidar sensors are dependent on lighting conditions of the scene, hence using just input from the Lidar can decrease robustness and become a serious issue when deploying to UAV. Nevertheless, comparing the performance of the current framework on different inputs (RGB, sparse depth, RGBd) is an interesting area of further research direction. We have already tested semi-dense depth interpolation (Makarov et al., 2019), however, fusion of sparse depth and hi-res RGB image is still a challenging task for real-time systems. We refer these directions for the future work.

##  Supplemental Information

10.7717/peerj-cs.317/supp-1 Supplemental Information 1 Code for experiments on KITTI dataset

## Additional Information and Declarations

### Data Availability

The following information was supplied regarding data availability: The code for training and metric evaluation for the conducted studies is available in the Supplemental Files. The KITTI dataset is available in: Geiger A, Lenz, P, Stiller C, Urtasun, R. (2013). Vision meets robotics: The KITTI dataset. International Journal of Robotics Research. http://www.cvlibs.net/datasets/kitti/raw_data.php.
